# Genus *Meleonoma* Meyrick, 1914 (Lepidoptera, Autostichidae) from Hainan Island, China, with descriptions of sixteen new species

**DOI:** 10.3897/zookeys.975.53289

**Published:** 2020-10-12

**Authors:** Xiaoju Zhu, Bo Cai, Shuxia Wang

**Affiliations:** 1 College of Life Sciences, Nankai University, Tianjin 300071, China; 2 Hainan Province Engineering Research Center for Quarantine, Prevention and Control of Exotic Pests, Haikou 570311, Hainan, China

**Keywords:** Distribution map, Gelechioidea, Microlepidoptera, morphology, taxonomy

## Abstract

Sixteen new species of the genus *Meleonoma* Meyrick, 1914 from Hainan Island, China are described: *M. apicicurvata* Wang, **sp. nov.**, *M. apicirectangula* Wang, **sp. nov.**, *M. bicuspidata* Wang, **sp. nov.**, *M. bidentata* Wang, **sp. nov.**, *M. conica* Wang, **sp. nov.**, *M. hainanensis* Wang, **sp. nov.**, *M. latiunca* Wang, **sp. nov.**, *M. linearis* Wang, **sp. nov.**, *M. magnidentata* Wang, **sp. nov.**, *M. ornithorrhyncha* Wang, **sp. nov.**, *M. parilis* Wang, **sp. nov.**, *M. pectinalis* Wang, **sp. nov.**, *M. puncticulata* Wang, **sp. nov.**, *M. quadritaeniata* Wang, **sp. nov.**, *M. robustispina* Wang, **sp. nov.** and *M. rostellata* Wang, **sp. nov.** Images of adult dorsal habitus and genitalia of the new species are provided. A map showing the collecting localities and photos of the habitat where the specimens were collected are provided, along with two maps showing the distribution of each species.

## Introduction

Meyrick (1914) described the genus *Meleonoma* and classified it in the family Oecophoridae. It was subsequently placed in Cosmopterigidae (Clarke 1965; Nye and Fletcher 1991; Li and Wang 2002, 2004), Lypusidae (Lvovsky 2015; Park and Park 2016), and back to Oecophoridae (Yin and Wang 2016a; Kitajima and Sakamaki 2019). In recent study, *Meleonoma* is hypothesized to be part of the subfamily Periacminae (Autostichidae) based on both molecular data and morphological study (Wang and Li 2020).

*Meleonoma* is characterized by having narrow to broad lanceolate forewings, the sacculus, in most species, separated from the valva entirely or distally, and a spinous patch on the tergites of both male and female. It is morphologically similar to the genus *Phaulolechia* Diakonoff, 1952, but can be distinguished by the termen of the forewing not concave below the apex, R_5_ extended to the costa, and CuA_1_ and CuA_2_ separate. In species of *Phaulolechia*, the termen of the forewing is concave below the apex, R_5_ extends to the termen, and CuA_1_ and CuA_2_ are fused at the base (Diakonoff 1952: 89).

*Meleonoma* is represented by 85 valid species (excluding five species with generic assignment uncertain) distributed in the Palearctic and Oriental regions (Wang et al. 2020; Wang and Tao 2020). Wang et al. (2020) proposed eight species groups for *Meleonoma* based on both molecular and morphological study, and assigned most described species to the proposed groups. The aim of the present paper is to report the species of *Meleonoma* collected on Hainan Island including the descriptions of sixteen new species.

**Figure 1.**
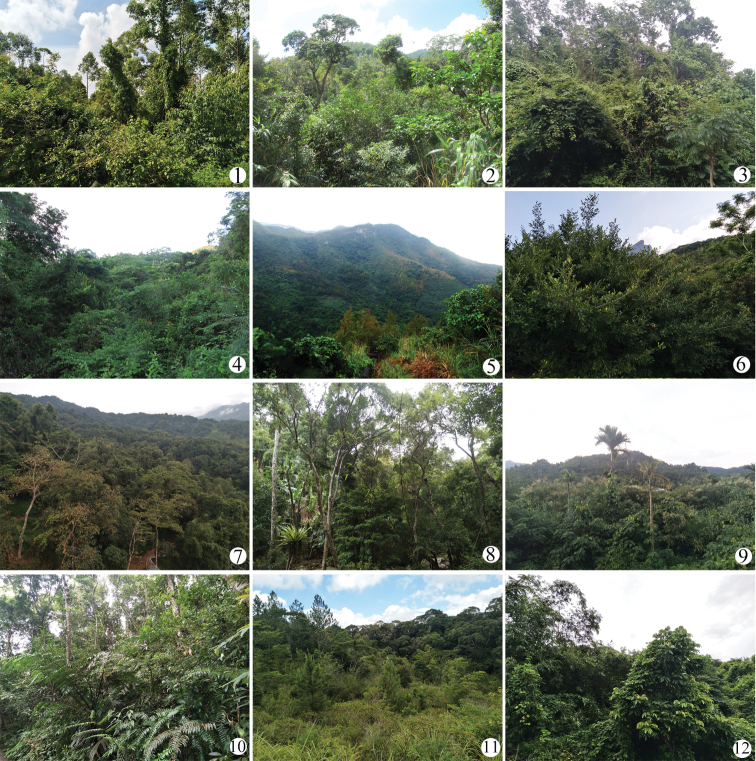
Ecological environment of collecting sites **1** Gaoshanling **2** Mt. Limu **3, 4** Bawangling **5, 6** Yinggeling **7, 8** Mt. Wuzhi **9** Mt. Diaoluo **10, 11** Jianfengling **12** Dali (Lingshui).

Hainan Island is located in the South China Sea east of Viet Nam and has an area of 34,000 square kilometers. It lies between tropical and subtropical regions, so has an average temperature between 22 °C and 26 °C. Hainan Island is rich in natural resources and biological diversity. There are 5860 species of plants known to occur on Hainan Island with 502 endemic species (Yang 2013), which provides a diversity of habitat for insects (Fig. 1).

The sixteen new species can be assigned to five species groups. *Meleonoma apicicurvata* sp. nov., *M. linearis* sp. nov. and *M. rostellata* sp. nov. belong to the *facialis* group, which are characterized by a yellow forewing with a dark costal spot and a dark apical patch. *Meleonoma magnidentata* sp. nov. belongs to the *acutiuscula* group, which are characterized by a dark forewing usually with two small yellow costal spots. *Meleonoma bicuspidata* sp. nov., *M. latiunca* sp. nov., *M. pectinalis* sp. nov., *M. hainanensis* sp. nov., *M. ornithorrhyncha* sp. nov., *M. parilis* sp. nov. and *M. quadritaeniata* sp. nov. belong to the *segregnatha* group, which share a dark forewing with more yellow spots. *Meleonoma bidentata* sp. nov. can be assigned to the *annulignatha* group, which are characterized by having a dark forewing with two yellow costal spots and a dorsal spot, an uncus with a bifurcate apex, and a circular gnathos. *Meleonoma apicirectangula* sp. nov. can be assigned to the *fasciptera* group, which are characterized by the forewing having a median yellow fascia and a yellow costal spot. *Meleonoma conica* sp. nov., *M. puncticulata* sp. nov. and *M. robustispina* sp. nov. share a yellow forewing with some small dark costal and terminal dots. We tentatively place these three species in a new group, the *puncticulata* group, which needs to be confirmed by molecular data.

In addition to the new species, we also collected and identified five described species in Hainan Island during this study: *M. apicispinata* Wang, 2016 and *M. liui* (Wang, 2006) belong to the *segregnatha* group; *M. facialis* Li & Wang, 2002 and *M. polychaeta* Li, 2004 belong to the *facialis* group; and *M. microbyrsa* (Wang, 2003) belongs to the *malacobyrsa* group.

**Figure 2.**
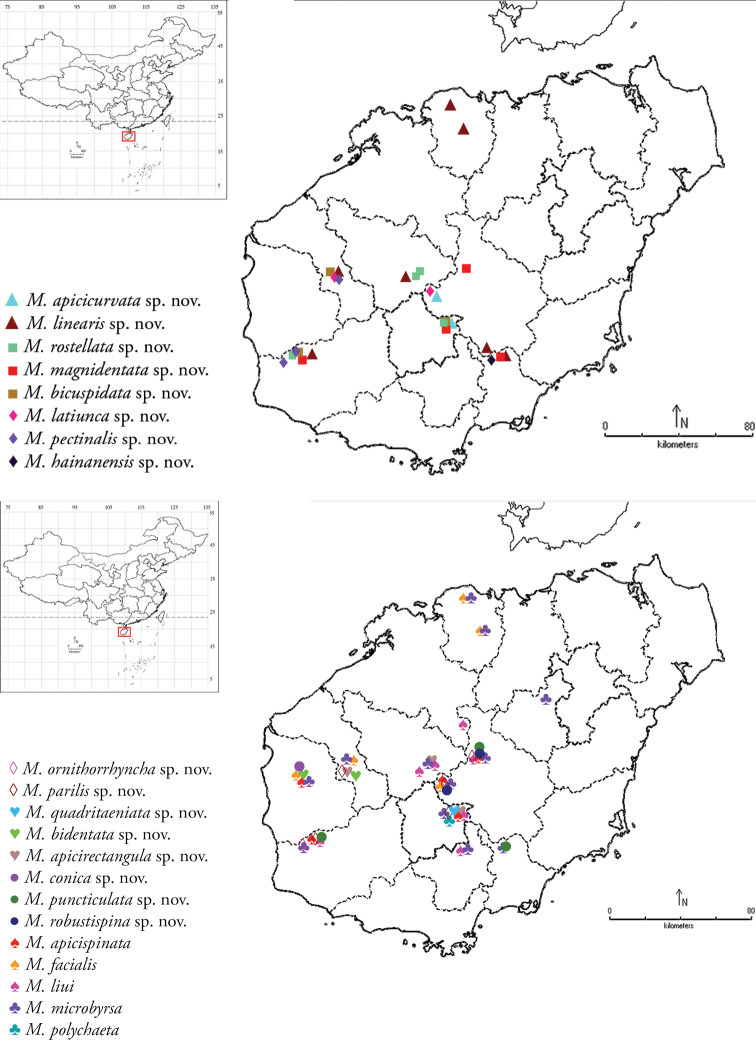
Distribution of the genus *Meleonoma* species in Hainan Island.

Two maps showing the localities of all 21 species collected on Hainan Island are provided (Fig. 2).

## Material and methods

**Figure 3.**
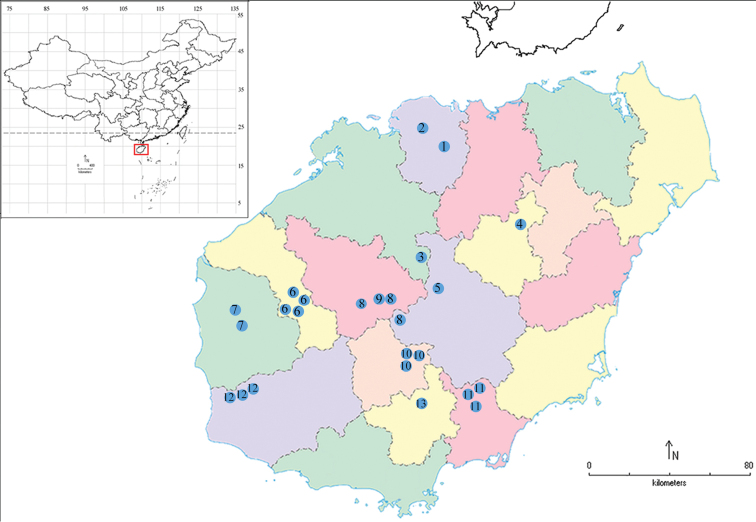
Collecting sites of *Meleonoma* species in Hainan Island **1** Duowenling **2** Gaoshanling **3** Danzhou **4** Mt. Wolong **5** Mt. Limu **6** Bawangling **7** Dongfang **8** Yinggeling **9** Baisha **10** Mt. Wuzhi **11** Mt. Diaoluo **12** Jianfengling **13** Qixianling.

The identification of species was based on dried specimens collected on Hainan Island from 2007 to 2018 by using light traps, with a small number being netted during daytime. We made 22 expeditions to this island, covering almost all the mountains of Hainan Island (Table 1; Fig. 3).

**Table 1.** Collecting information.

Localities	Latitude and longitude	Date	Collectors
Duowenling	19.79N, 109.76E	VIII.2017	X Bai et al.
Gaoshanling	19.93N, 109.64E	VIII.2017, VI.2018	X Bai et al., P Liu et al.
Danzhou	19.32N, 109.69E	XII.2017	MJ Qi et al.
Mt. Wolong	19.46N, 110.12E	VI.2018	P Liu et al.
Mt. Limu	19.17N, 109.73E	IV.2008, V.2014, VII.2014, V.2015, VI.2015, I.2016, X.2016	BB Hu et al., TT Liu et al., PX Cong et al., KJ Teng et al., X Bai et al.
Bawangling	19.07N, 109.03E	IV.2008, IV.2009	BB Hu et al.
	19.10N, 109.11E	V.2013, VII.2013, VIII.2017, VI.2018	YH Sun et al., HL Yu et al., X Bai et al., P Liu et al.
	19.12N, 109.08E	VII.2014, VI.2015, III.2016	PX Cong et al., QY Wang et al.
	19.08N, 109.10E	VII.2015	QY Wang et al.
Dongfang	19.11N, 108.79E	XII.2009	ZH Du et al.
	19.04N, 108.84E	I.2018, VI.2018	MJ Qi et al., P Liu et al.
Yinggeling	19.03N, 109.55E	VI.2010, IX.2010. X.2013,	BB Hu et al., ZB Wang et al.
	19.08N, 109.50E	VI.2014, I.2016, III.2016, VII‒VIII.2017	PX Cong et al., KJ Teng et al., QY Wang et al., X Bai et al.
	19.07N, 109.40E	XI.2016	X Bai et al.
Baisha	19.07N, 109.52E	IV.2014, VII.2014, VI.2015, VII.2015, III.2016	TT Liu et al., PX Cong et al., QY Wang et al.
Mt. Wuzhi	18.90N, 109.67E	V.2007, IV.2009, V.2010, VII.2014, VII.2015, II.2016, III.2016, VII.2016, X‒XI.2016, VII.2017	ZW Zhang et al., BB Hu et al., Q Jin et al., PX Cong et al., QY Wang et al., X Bai et al.
	18.91N, 109.68E	IV.2014	TT Liu et al.
	18.91N, 109.70E	VIII.2017	X Bai et al.
Mt. Diaoluo	18.73N, 109.87E	V‒VI.2007, IV.2014, V.2015	ZW Zhang et al., TT Liu et al., PX Cong et al.
	18.72N, 109.86E	VII.2014	PX Cong et al.
	18.67N, 109.92E	VII.2017	X Bai et al.
Jianfengling	18.75N, 108.87E	VI.2007, IV.2008, VII.2014, V.2015, VII.2015, III.2016, VIII.2016, VIII.2017, VI.2018	ZW Zhang et al., BB Hu et al., PX Cong et al., QY Wang et al., X Bai et al., P Liu et al.
	18.74N, 108.84E	VI.2010, VIII.2017	BB Hu et al., X Bai et al.
	18.70N, 108.79E	IV‒V.2013, IV.2014	YH Sun et al., TT Liu et al.
Qixianling	18.70N, 109.67E	IV.2013, XII.2017	YH Sun et al., MJ Qi et al.

Genitalia dissection and mounting follow the methods introduced by Li (2002), stained using Eosin Y, occasionally with Chlorazol Black E for membranous structure. Images of adults were taken with a Leica M205A stereomicroscope and genitalia were prepared using a Leica DM750 microscope, equipped with Leica Application Suite 4.2 software. Distribution maps were prepared using DIVA-GIS Ver. 7.5.0 and output as TIF files that were edited subsequently in Adobe Photoshop CC.

Terminology follows Wang et al. (2020). Species are arranged by species groups.

The type specimens are deposited in the Insect Collection of Nankai University, Tianjin, China (**NKU**).

## Taxonomy

### *Meleonoma* Meyrick, 1914

*Meleonoma* Meyrick, 1914: 255. Type species: *Cryptolechia stomota* Meyrick, 1910: 224.

*Acryptolechia* Lvovsky, 2010: 255. Type species: *Cryptolechia malacobyrsa* Meyrick, 1921: 394.

### The *facialis* species group

#### Meleonoma apicicurvata

Taxon classificationAnimaliaLepidopteraCosmopterigidae

Wang sp. nov.

12882980-9A50-51A1-9853-3C65AA06F5ED

http://zoobank.org/56174C79-42FE-4651-9300-B5797BC72A32

Figs 4 , 20

##### Type material.

China, Hainan: ***Holotype*** ♂, Yinggezui (19.05N, 109.56E), Yinggeling, 599 m, 30.VII.2017, leg. X Bai et al., slide No. ZXJ19011. ***Paratype***: 1♂, Mt. Wuzhi, 732 m, 4.VIII.2017, leg. X Bai et al.

##### Diagnosis.

The new species can be distinguished from its congeners by having the apex of the valva curved dorsally at almost a right angle, a sub-ovate process extending from beyond the sacculus of the valva, and a large vinculum extended to a broadly rounded anterior margin.

##### Description.

**Figures 4–11.**
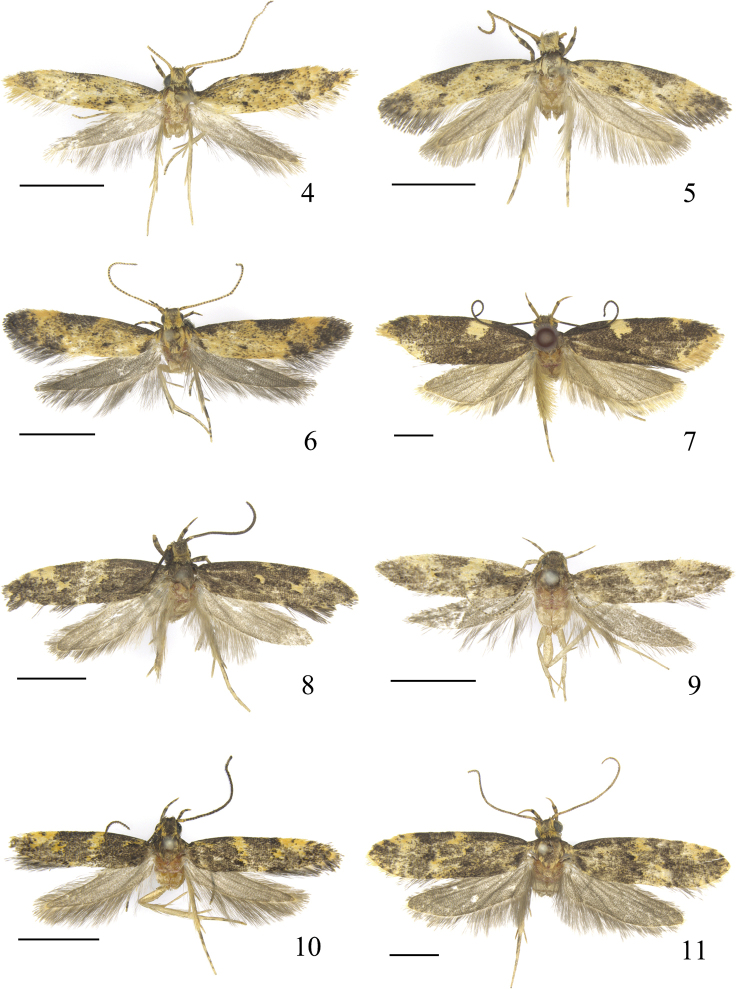
Male adults of *Meleonoma* spp. **4***M. apicicurvata* sp. nov. **5***M. linearis* sp. nov. **6***M. rostellata* sp. nov. **7***M. magnidentata* sp. nov. **8***M. bicuspidata* sp. nov. **9***M. latiunca* sp. nov. **10***M. pectinalis* sp. nov. **11***M. hainanensis* sp. nov. All holotypes. Scale bars: 2.0 mm.

Adult (Fig. 4). Wingspan 8.0‒10.0 mm. Head yellow, vertex mixed with black. Labial palpus yellow; second segment with distal 2/3 mixed with black scales, forming a black ring at apex; third segment mixed with black scales from middle to before apex on dorsal surface. Antenna yellow; flagellum alternated with black on dorsal surface except several basal flagellomeres yellow. Thorax yellow; tegula black basally, yellow distally. Forewing yellow, with black scales; costal margin with basal 1/4 black, diffused to above fold posteriorly; costal spot black, sub-triangular, from between basal 2/5 and 2/3 extending crossing anterior margin of cell posteriorly; apical patch black, running from distal part of costal margin along termen; tornal spot black, ill-defined; distal and plical spots black, ovate; black dot at anterior angle of cell distinct; fringe yellow, tinged with blackish brown. Hindwing and fringe grey. Legs yellow, with exception on ventral surface: foreleg mixed with black scales on femur and tibia, first tarsomere of tarsus with a black dot, apical three tarsomeres black; midleg with femur black apically, tibia black except yellow apically, tarsus black except yellow at apices of basal two tarsomeres; hindleg with scattered black scales.

**Figures 20–27.**
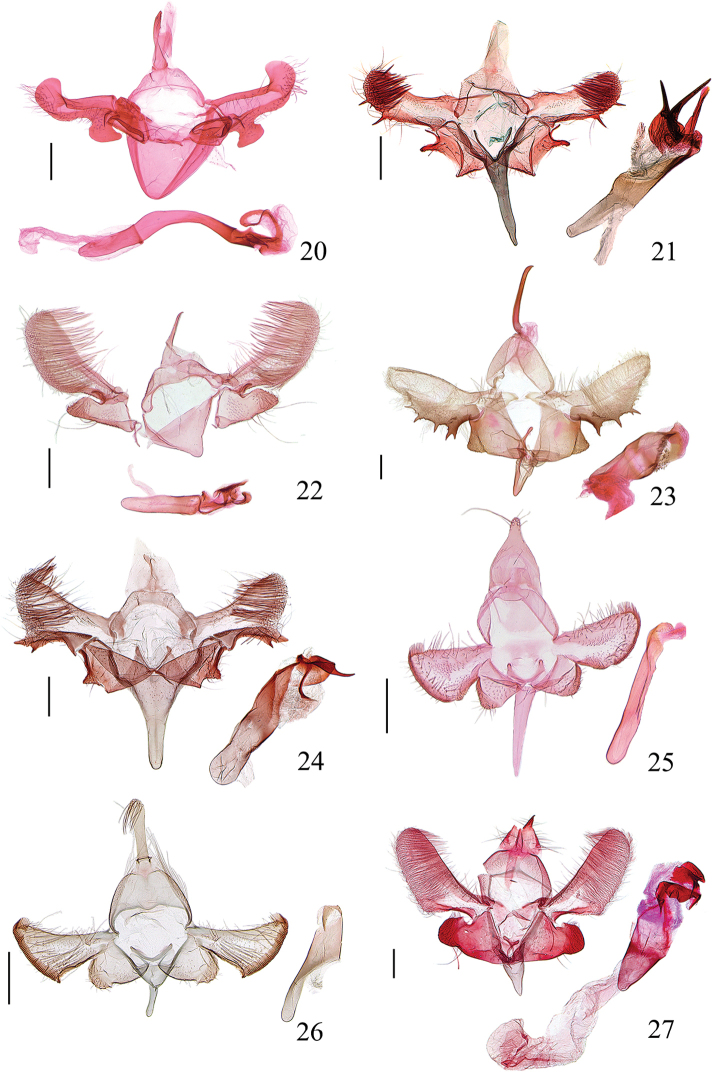
Male genitalia of *Meleonoma* spp. **20***M. apicicurvata* sp. nov., slide No. ZXJ19011 **21***M. linearis* sp. nov., slide No. YAH15402 **22***M. rostellata* sp. nov., slide No. ZXJ18130 **23***M. magnidentata* sp. nov., slide No. LJ17530 **24***M. bicuspidata* sp. nov., slide No. YAH15447 **25***M. latiunca* sp. nov., slide No. ZXJ18430 **26***M. pectinalis* sp. nov., slide No. YAH15502 **27***M. hainanensis* sp. nov., slide No. YAH15508. All holotypes. Scale bars: 0.2 mm.

***Male genitalia*** (Fig. 20). Uncus slightly wide at base, narrowed from base to pointed apex. Tegumen widened medially; lateral arm slightly narrowed anteriorly. Valva wide at base, slightly narrowed from base to before basal 2/3, curved upward at ca. distal 1/3 almost at a right angle, obtusely rounded at apex; ventral margin heavily sclerotized, forming a wide band reaching ca. distal 1/3, with a large sub-ovate process beyond sacculus; costa wide, with sparse setae; transtilla short, narrowed to pointed apex. Sacculus sub-triangular, relatively small, heavily sclerotized on margins, with sparse long setae on dorsal surface. Vinculum extended anteriorly, broadly rounded on anterior margin. Juxta slender, arched in U-shape (attached to apex of phallus). Phallus slender, strongly curved medially, more than twice length of valva, heavily sclerotized distally, with a fusiform sclerite.

##### Female

unknown.

##### Distribution.

Hainan (Mt. Wuzhi, Yinggeling).

##### Etymology.

The specific epithet is derived from the Latin *apic*- (adj., apical) and *curvatus* (adj., curved), referring to the valva curved dorsally at almost a right angle in the male genitalia.

#### Meleonoma linearis

Taxon classificationAnimaliaLepidopteraCosmopterigidae

Wang sp. nov.

6741FFA3-5EE4-5CFE-A1B5-8F04A76A6AD6

http://zoobank.org/EE18FC98-AB59-42A0-B813-3E2ECD0C94D9

Figs 5 , 21

##### Type material.

China, Hainan: ***Holotype*** ♂, Tianchi (18.73N, 108.87E), Jianfengling, 787 m, 13.VII.2015, leg. QY Wang et al., slide No. YAH15402. ***Paratypes*** (15♂): 1♂, 12.VII.2015, other same data as holotype; 5♂, Tianchi, Jianfengling, 787 m, 5‒8.III.2016, leg. QY Wang et al.; 1♂, Duowenling, 207 m, 20.VIII.2017, leg. X Bai et al.; 3♂, Gaoshanling, 171 m, 18‒19.VIII.2017, leg. X Bai et al.; 1♂, Bawangling, 146 m, 16.VIII.2017, leg. X Bai et al.; 1♂, Nankai, Yinggeling, 270 m, 8.XI.2016, leg. X Bai et al.; 1♂, Dali, Lingshui, 229 m, 24.VIII.2017, leg. X Bai et al.; 2♂, Mt. Diaoluo, 84 m, 22.VII.2017, leg. X Bai et al.

##### Diagnosis.

The new species can be distinguished from its congeners by the ventral margin of the valva with a spine apically, the rectangular sacculus with a longer digitate dorsoapical process and a small triangular ventroapical process, and the phallus with two stout spine-shaped cornuti. It is similar to *M. triangula* Wang, 2016 superficially, but can be distinguished by the valva subparallel from base to apex, the lineate transtilla, and the phallus with two cornuti. In *M. triangula*, the valva is widened distally and produced dorsoapically, the transtilla is triangular, and the phallus lacks a cornutus (Yin and Wang 2016b: 135, fig. 6).

##### Description.

Adult (Fig. 5). Wingspan 9.5‒10.0 mm. Head yellow, in some individuals, head blackish brown, yellow along dorsal margin of eyes and occiput. Labial palpus yellow; second segment with dense blackish brown scales on outer surface, forming a ring at apex; third segment ca. 1/2 length of second segment, with dense blackish brown scales. Antenna yellow; scape blackish brown basally; flagellum alternated with blackish brown on dorsal surface. Thorax blackish brown, pale yellow laterally; tegula blackish brown basally, pale yellow distally. Forewing pale yellow, with scattered blackish brown scales; with a rounded black spot at base below costal margin, with an ovate blackish brown spot near base of cell; costal margin blackish brown along basal 1/3; costal spot blackish brown, large, inverted triangular, from between basal 1/3 and basal 2/3 extending crossing anterior margin of cell posteriorly; apical patch blackish brown; tornal spot greyish black, small; discal and plical spots blackish brown; blackish brown dot at anterior and posterior angles of cell, touching costal and tornal spots respectively; dorsum with a diffused blackish brown spot at base; fringe greyish black. Hindwing and fringe grey. Legs yellow, with exception on ventral surface: fore tarsus blackish brown, yellow at apices of basal two tarsomeres, tarsi of mid- and hindlegs blackish brown except yellow at apex of each tarsomere; all tibiae blackish brown except yellow apically.

***Male genitalia*** (Fig. 21). Uncus slightly wide at base, narrowed from base to rounded apex. Tegumen U-shaped, widened medially; lateral arm uniformly wide. Valva subparallel from base to apex, with dense long and stout setae distally; apex obtusely rounded, with a spine arising from above ventroapical corner; ventral margin sclerotized, forming a narrow band ending with a long spine apically, with a small sub-triangular process at base; costa convex and sparsely setose basally, shallowly concave medially; transtilla lineate, straight inwards, shortly joined by membrane. Sacculus rectangular, shorter than width; dorsal margin narrowly sclerotized, concave before apex; apex shallowly concave, produced to a clavate dorsoapical process and a small triangular ventroapical process, with a fold from ventroapical process extending obliquely upward to ca. middle of sacculus. Saccus clavate, ca. twice as long as uncus, wide at base, narrowed from base to rounded apex. Juxta V-shaped. Phallus approximately 1.5 × length of valva, widened medially, partly membranous distally, with a straightly clubbed process apically; cornuti being two large stout spines, placed distally.

##### Female

unknown.

##### Distribution.

Hainan (Bawangling, Duowenling, Gaoshanling, Jianfengling, Lingshui, Mt. Diaoluo, Yinggeling).

##### Etymology.

The specific epithet is derived from the Latin *linearis* (adj., lineate), referring to the lineate transtilla in the male genitalia.

#### Meleonoma rostellata

Taxon classificationAnimaliaLepidopteraCosmopterigidae

Wang sp. nov.

A310EF7D-C22F-5C24-B336-A02F9793598E

http://zoobank.org/A75EF0C9-334E-43E2-9EBE-448BC1D9DE57

Figs 6 , 22 , 36

##### Type material.

China, Hainan: ***Holotype*** ♂, Hongkan (19.08N, 109.50E), Yinggeling, 540 m, 20.I.2016, leg. KJ Teng et al., slide No. ZXJ18130. ***Paratypes*** (17♂15♀): 3♀, Hongkan, Yinggeling, 540 m, 15.III.2016, leg. QY Wang et al., slide No. ZXJ19410; 1♂, Hongxin, Yuanmen, Baisha, 445 m, 3.VIII.2015, leg. QY Wang et al.; 3♂2♀, Mt. Wuzhi, 742 m, 6‒8.VII.2014, leg. PX Cong et al.; 6♂7♀, 5‒8.VII.2015, 1♂1♀, 27.II.2016, Lizudadian, Shuiman, Mt. Wuzhi, 766 m, leg. QY Wang et al.; 4♂2♀, 738 m, Mt. Wuzhi, 3.III.2016, leg. QY Wang et al.; 2♂, Tianchi, Jianfengling, 11.VI.2010, leg. BB Hu & J Zhang.

##### Diagnosis.

The new species is similar to *M. dorsolobulata* Wang, 2016 in the male genitalia, but can be differentiated from the latter by the valva with a small rostral process at base on the ventral margin, the sacculus serrate and obliquely truncate apically, and the saccus shorter than the uncus; in *M. dorsolobulata*, the valva has a lobate process at base on the ventral margin, the sacculus is obliquely rounded dorsoapically, and the saccus is as long as the uncus (Yin and Wang 2016b: 136, fig. 7). The new species can be distinguished in the female genitalia by the lamella antevaginalis concave in V-shape medially on the anterior margin that forms two rounded reticulate anterolateral lobes, and the corpus bursae with two signa different in size.

##### Description.

Adult (Fig. 6). Wingspan 7.0‒8.0 mm. Head with frons yellow; vertex blackish grey, with yellow scales laterally; occiput yellow. Labial palpus yellow; second segment with scattered blackish grey scales on outer surface, with a blackish grey ring apically; third segment blackish grey medially, ca. 2/3 length of second segment. Antenna yellow, scape mixed with blackish brown scales dorsally; flagellum ringed with brown on dorsal surface. Thorax blackish brown medially, yellow laterally; tegula blackish brown basally, yellow distally. Forewing yellow, with scattered black scales; costal margin with a black stripe along basal 1/3, widened basally, with a small dim black dot before apex; costal spot black, large, semicircular, extending crossing anterior margin of cell posteriorly, slightly placed beyond middle; apical patch black, large; tornal spot black, diffused to apical patch along termen, before tornal spot situated a black dot; discal and plical spots black; black dot at anterior and posterior angles of cell respectively, almost inseparable from costal and tornal spots; dorsum with a black spot at base; fringe greyish black. Hindwing and fringe blackish grey. Legs yellow, with exception on ventral surface: foreleg mixed with blackish grey scales on coxa, tarsi of fore- and midlegs blackish grey except yellow at apices of basal two tarsomeres, tarsus of hindleg yellow at apex of each tarsomere; all femora mixed with blackish grey scales.

***Male genitalia*** (Fig. 22). Uncus wide at base, narrowed to hooked apex. Tegumen broad U-shaped, widened medially; lateral arm almost uniform, sclerotized along outer and inner margins, rounded anteriorly. Valva narrow at base, subparallel from basal 1/4 to before rounded apex, densely setose; ventral margin sclerotized along basal 1/4, forming a narrow band, with a small rostral process at base; costa band-shaped, reaching basal 2/5 of valva, with sparse setae basally; transtilla ovately dilated. Sacculus wide at base, weakly narrowed to apex, densely setose in distal 1/4; apex obliquely truncate, finely serrate; dorsal margin concave at middle; ventral margin sclerotized, forming a narrow band, weakly serrate from basal 1/6 to middle, with sparse long setae in distal half. Vinculum extended anteriorly, forming a small papillary saccus. Saccus shorter than uncus. Juxta slender. Phallus slightly longer than valva, basal 3/5 tubular, distal 2/5 partly membranous, with several curved, irregularly shaped belts.

***Female genitalia*** (Fig. 36). Papillae anales sub-quadrate, setose. Apophyses posteriores approximately 2.5 × as long as apophyses anteriores. Eighth sternal plate spiculate; posterior margin bearing long setae, deeply and narrowly incised medially, forming two large semicircular lateral lobes; anterior margin convex medially. Lamella antevaginalis straight on posterior margin, concave in V-shape medially on anterior margin, forming two rounded reticulate anterolateral lobes. Ductus bursae membranous, widened anteriorly; ductus seminalis arising from ductus bursae near entrance of corpus bursae. Corpus bursae rounded, nearly as long as ductus bursae, with two signa different in size: one sub-rounded, placed at entrance of corpus bursae, with dense teeth, the other elongate-ovate, with dense teeth and a large spine.

##### Distribution.

Hainan (Baisha, Jianfengling, Mt. Wuzhi, Yinggeling).

##### Etymology.

The specific epithet is derived from the Latin *rostellatus* (adj., rostrated), referring to the rostral process at the base on the ventral margin of the valva in the male genitalia.

### The *acutiuscula* species group

#### Meleonoma magnidentata

Taxon classificationAnimaliaLepidopteraCosmopterigidae

Wang sp. nov.

7A1CEFF0-8876-522F-BD0C-AC9CC4830738

http://zoobank.org/489650A4-EC5B-49EE-A629-8F16824C32A0

Figs 7 , 23 , 37

##### Type material.

China, Hainan: ***Holotype*** ♂, Tianchi (18.73N, 108.87E), Jianfengling, 787 m, 10.III.2016, leg. QY Wang et al., slide No. LJ17530. ***Paratypes*** (4♂3♀): 1♂, Forest Park, Mt. Limu, 607 m, 15.V.2015, leg. PX Cong et al.; 1♂1♀, Mt. Wuzhi, 738 m, X.29‒XI.4.2016, leg. X Bai et al., slide No. ZXJ18261♀; 1♂, Mt. Wuzhi, 732 m, 2.VIII.2017, leg. X Bai et al.; 1♂1♀, Mt. Diaoluo, 940 m, 31.V‒2.VI.2007, leg. ZW Zhang & WC Li; 1♀, Mt. Diaoluo, 922 m, 24.V.2015, leg. PX Cong et al.

##### Diagnosis.

The new species can be distinguished from its congeners by the valva with several large, strong denticles different in number and size on the ventral margin, and by the lamella antevaginalis being broad rectangular ventrally, narrowly banded dorsally, and joined laterally.

##### Description.

Adult (Fig. 7). Wingspan 18.0‒18.5 mm. Head blackish brown, mixed with yellow. Labial palpus yellow; second segment with blackish brown scales on outer surface, with a blackish brown ring at apex; third segment slightly shorter than second segment, mixed with sparse blackish brown scales. Antenna with scape blackish brown basally, yellow distally; flagellum blackish brown. Thorax and tegula blackish brown. Forewing blackish brown; costal margin with two yellow spots: antemedian spot rectangular, from before basal 1/3 oblique outwards, crossing anterior margin of cell posteriorly, distal spot sub-triangular, at ca. distal 1/4; cell with a small yellow spot near outer margin; plical spot yellow, very small; fringe yellow, except greyish black along distal part of costal margin and around tornus. Hindwing and fringe yellowish grey. Legs yellow, with exception on ventral surface: foreleg blackish brown, femur scattered with yellow scales apically, tarsus yellow at apices of basal two tarsomeres; mid- and hindlegs blackish brown, tarsi yellow at apex of each tarsomere; all tibiae yellow apically.

***Male genitalia*** (Fig. 23). Uncus clavate, uniformly wide from near base to before pointed apex. Gnathos sclerotized laterally, exceeding anterior margin of tegumen, membranous anteriorly. Tegumen V-shaped, uniformly wide, with sclerotized edges. Valva narrow at base, widened from base to basal 1/3, thereafter narrowed to rounded apex, setose; ventral margin projected medially, with several large, strong denticles along basal 2/3, different in number and size on left and right valvae; costa lineate, reaching before apex of valva, with sparse setae basally; transtilla short and wide, narrowed to apex, with long setae at base. Sacculus wide at base, narrowed from base to before apex; apex triangularly produced, setose; ventral margin sclerotized and folded. Saccus ca. 1/2 length of uncus, wide at base, narrowed to rounded apex. Juxta U-shaped; lateral lobe clubbed, slightly enlarged distally. Phallus stout, approximately as long as valva, slightly widened medially, with a spine before apex and numerous teeth sparsely grouped below it.

***Female genitalia*** (Fig. 37). Papillae anales sub-rectangular, setose. Apophyses posteriores ca. 2.5 × length of apophyses anteriores. Eighth sternal plate with posterior margin rounded, slightly concave at middle, lined with sparse long setae. Lamella antevaginalis ventrally broad rectangular, concave medially on anterior margin, narrowly banded dorsally, joined laterally. Ductus bursae with posterior 3/4 membranous, uniformly wide, anterior 1/4 sclerotized; ductus seminalis arising from anterior 1/3. Corpus bursae as long as ductus bursae, widened anteriorly; signum absent.

##### Distribution.

Hainan (Jianfengling, Mt. Limu, Mt. Diaoluo, Mt. Wuzhi).

##### Etymology.

The specific epithet is derived from the Latin *magni*- (adj., large) and *dentatus* (adj., dentate), referring to the large denticles on the ventral margin of the valva.

### The *segregnatha* species group

#### Meleonoma bicuspidata

Taxon classificationAnimaliaLepidopteraCosmopterigidae

Wang sp. nov.

F2CB0203-9A30-5D9F-94B6-D39F883293A5

http://zoobank.org/69D700C8-9B21-42D9-B359-C962E0143025

Figs 8 , 24

##### Type material.

China, Hainan: ***Holotype*** ♂, Mt. Wuzhi (18.90N 109.67E), 742 m, 8.VII.2014, leg. PX Cong et al., slide No. YAH15447. ***Paratypes*** (3♂): 1♂, Bawangling, 161 m, 7.VI.2015, leg. PX Cong et al.; 1♂, Jianfengling, 770 m, leg. 31.V.2015, PX Cong et al.; 1♂, Tianchi, Jianfengling, 787 m, 16.VII.2015, leg. QY Wang et al.

##### Diagnosis.

The new species can be distinguished from its congeners by the uniformly wide tegumen straight on posterior margin, the sclerotized and widely banded ventral margin of the valva with two apical spines close at base, and the sacculus with a papillary process below middle of apex.

##### Description.

Adult (Fig. 8). Wingspan 10.0‒11.0 mm. Head greyish black, yellow laterally, occiput yellow tipped with greyish black. Labial palpus yellow; second segment mixed with dense black scales on outer surface, forming a black ring at apex; third segment shorter than second segment, with blackish grey scales in basal 2/3. Antenna with scape greyish black basally, yellow distally; flagellum greyish black, annulated with yellow on ventral surface. Thorax and tegula greyish black. Forewing greyish black; costal margin with median yellow spot before middle, small, distal yellow spot at distal 1/4, large, inverted triangular, extending crossing anterior angle of cell posteriorly, with a greyish black dot anteromedially; discal spot black, rounded, with crescent yellow spot encircled its anterior and outer margins; plical spot black, placed at distal 1/3, edged with yellow scales; cell with two black spots near outer margin, placed one above the other, with a large yellow spot between them; dorsal spot yellow, small, placed at end of fold; fringe greyish black mixed with yellow. Hindwing and fringe pale grey. Legs yellow, with exception on ventral surface: fore coxa greyish black, tarsi of fore- and midlegs greyish black, yellow at apices of basal two tarsomeres, hind tarsus greyish black, yellow at apex of each tarsomere; all tibiae greyish black, yellow apically.

***Male genitalia*** (Fig. 24). Uncus wide at base, narrowed from base to ca. middle, uniformly narrow from middle to narrowly rounded apex. Gnathos sclerotized laterally, not exceeding posterior margin of tegumen, invisible anteriorly. Tegumen uniformly wide except lateral arm slightly narrowed anteriorly, straight on posterior margin, distinctly angled posterolaterally. Valva narrow basally, widened distally; apex obliquely obtuse, produced dorsoapically, with dense setae; ventral margin heavily sclerotized, forming a wide band, triangularly projected at base, concave medially, with two short apical spines close at base: dorsal spine large and narrowly rounded at apex, ventral spine short, pointed at apex; costa wide basally, slightly narrowed distally, reaching apex of valva; transtilla shortly enlarged, not extended. Sacculus wide at base, narrowed to apex; apex oblique, with a sclerotized edge along posterior half, with a setose papillary process below middle; ventral margin overlapped triangularly. Saccus more than twice length of uncus, narrowed from broad base to middle, subparallel from middle to before rounded apex. Juxta arched in V-shape, inflated apically. Phallus slightly longer than valva; basal half tubular, sclerotized, distal half partly membranous; narrow ribbon-like belt crossing beyond distal 1/4, then one branch curved and extending downward to before apex; the other branch extending outward, slender basally, widened from basal 1/3 to distal 1/3, fused with apex of phallus, being a large free spine distally.

##### Female

unknown.

##### Distribution.

Hainan (Bawangling, Jianfengling, Mt. Wuzhi).

##### Etymology.

The specific epithet is derived from the Latin *bicuspidatus* (adj., having two spines), referring to the two apical spines on the ventral margin of the valva.

#### Meleonoma latiunca

Taxon classificationAnimaliaLepidopteraCosmopterigidae

Wang sp. nov.

49B708C0-5426-5B6E-A8BB-3280973389F0

http://zoobank.org/91847E42-3F86-4FAD-B28C-7E83EE14118E

Figs 9 , 25

##### Type material.

China, Hainan: ***Holotype*** ♂, Yinggezui (19.05N, 109.56E), Yinggeling, 599 m, 30.VII.2017, leg. X Bai et al., slide No. ZXJ18430. ***Paratypes*** (2♂): 1♂, 31.VII.2017, other same data as holotype; 1♂, Bawangling, 146 m, 16.VIII.2017, leg. X Bai et al.

##### Diagnosis.

The new species can be distinguished from its congeners by the uncus with several long stout setae distally and the valva with a row of short setae arranged like a comb apically. It is similar to *M. pectinalis* sp. nov., and the differences between them are stated in the diagnosis of the latter species.

##### Description.

Adult (Fig. 9). Wingspan 8.0‒10.0 mm. Head greyish brown. Labial palpus yellow; first and second segments with dense blackish grey scales; second segment with two indistinct blackish brown rings in distal half; third segment shorter than second segment, with scattered blackish grey scales. Antenna yellow; scape with dense greyish black scales dorsally; flagellum annulated with blackish grey on dorsal surface. Thorax and tegula blackish grey. Forewing broad lanceolate, apex narrowly rounded; ground color blackish grey, with scattered black scales, with a rounded yellow spot at base below costal margin; costal margin with median yellow spot rectangular, from basal 2/5 extending obliquely outward to before posterior margin of cell, distal yellow spot elongate elliptical, from distal 1/4 extending to anterior angle of cell; cell with black spot at ca. basal 2/3 and at posterior angle respectively, edged with yellow scales; plical spot black, edged with yellow scales; dorsal yellow spot at end of fold, smaller; fringe blackish grey. Hindwing and fringe pale greyish brown. Legs greyish yellow; on ventral surface, foreleg mixed with greyish brown on coxa, tarsi of fore- and midlegs greyish brown, yellow at apices of basal two tarsomeres, hind tarsus greyish brown, yellow at apex of each tarsomere; all femora mixed with greyish brown scales, tibiae greyish brown except yellow apically.

***Male genitalia*** (Fig. 25). Uncus wide at base, narrowed from base to basal 2/3, outer margin convex; distal 1/3 uniformly wide except narrowly rounded apex, with several stout setae. Gnathos sclerotized laterally, membranous anteriorly. Tegumen narrowed medially; lateral arm uniformly narrow, rounded anteriorly. Valva sub-triangular, narrow at base, widened to rounded apex; apex with a row of short setae arranged like a comb, running from pre-apex of costa along apex to ventroapex, obliquely rounded; ventral margin heavily sclerotized, with a crescent sclerite at base; costa straight, with sparse setae. Sacculus wide at base, slightly narrowed to obtuse apex; apex heavily sclerotized, forming a sclerotized band. Saccus slender, slightly longer than uncus, wide at base, narrowed from base to narrowly rounded apex. Juxta broadly U-shaped; lateral lobe short. Phallus longer than valva, tubular; cornutus absent.

**Female** unknown.

##### Distribution.

Hainan (Bawangling, Yinggeling).

##### Etymology.

The specific epithet is derived from the Latin *latus* (adj., broad) and the Latin term *uncus* (n., uncus), referring to the basally wide uncus.

#### Meleonoma pectinalis

Taxon classificationAnimaliaLepidopteraCosmopterigidae

Wang sp. nov.

9951EFE5-05BB-5D34-8A6E-0B0AAA8B4D3E

http://zoobank.org/70354FC1-D460-4EDD-AF75-FB746AFB50E3

Figs 10 , 26 , 38

##### Type material.

China, Hainan: ***Holotype*** ♂, Tianchi (18.73N, 108.87E), Jianfengling, 787 m, 15.VII.2015, leg. QY Wang et al., slide No. YAH15502. ***Paratypes*** (33♂4♀): 1♂, same data as holotype; 1♂, Tianchi, Jianfengling, 787 m, 8.III.2016, leg. QY Wang et al.; 3♂, Tianchi, Jianfengling, 787 m, 5.VIII.2016, leg. X Bai et al.; 1♂1♀, Jianfeng, 40 m, 25.IV.2014, leg. TT Liu et al., slide No. YAH15095♀; 1♂, Jianfeng, 40 m, 28.IV.2013, leg. YH Sun et al.; 20♂, Jianfengling, 770 m, 13‒17.VII.2014, leg. PX Cong et al.; 1♂, Jianfengling, 745 m, 7.VIII.2017, leg. X Bai et al.; 1♂, Mingfenggu, Jianfengling, 954 m, 10.VIII.2017, leg. X Bai et al.; 1♂1♀, Jianfengling, 810 m, 12‒14.VI.2018, leg. P Liu et al.; 1♂, Bawangling, 161 m, 20.VII.2014, leg. PX Cong et al.; 1♂, Bawangling, 161 m, 12.III.2016, leg. QY Wang et al.; 1♂2♀, Bawangling, 225 m, 12‒14.VIII.2017, leg. X Bai et al.

##### Diagnosis.

The new species can be distinguished from its congeners by the uncus with a tuft of long setae at apex, and by the elliptical corpus bursae full of dense denticles that form a conspicuous inverted U-shaped area. It is similar to *M. latiunca* sp. nov. in the male genitalia, but can be distinguished by the uncus dilated distally, the tegumen widened medially, and the saccus slightly shorter than the uncus; in *M. latiunca*, the uncus is not dilated distally, the tegumen is narrowed medially, and the saccus is longer than the uncus.

##### Description.

Adult (Fig. 10). Wingspan 8.0‒9.0 mm. Head black, vertex with yellow scales laterally. Labial palpus yellow; second segment with dense blackish brown in basal 2/3, with a blackish brown ring before apex; third segment nearly 2/3 length of second segment, with blackish brown scales medially. Antenna with scape yellow, mixed with blackish brown scales; flagellum blackish brown, ringed with yellow ventrally. Thorax greyish black, mixed with sparse yellow scales; tegula greyish black. Forewing greyish black, with a diffused yellow spot at base below costal margin; costal margin with two orange spots: first spot from basal 1/3 extending to middle of cell posteriorly, sub-rectangular, second spot triangular, from distal 1/4 extending obliquely to beyond anterior angle of cell posteriorly, with a blackish brown dot at middle anteriorly; cell with a black spot at basal 1/3, with two black spots near outer margin placed one above the other, with a large orange spot between them, small black dots on inside of yellow spot; plical spot black, rounded, bordered by orange-yellow spots on outside, placed at distal 1/3 of fold; dorsal spot orange, smaller, placed before end of fold; fringe greyish black, mixed with yellow basally. Hindwing and fringe greyish brown. Legs yellow, with exception on ventral surface: fore coxa blackish brown, tarsi of fore- and midlegs blackish brown, yellow at apices of basal two tarsomeres, hind tarsus blackish brown, yellow at apex of each tarsomere; all femora with dense blackish brown scales, tibiae blackish brown, yellow apically.

***Male genitalia*** (Fig. 26). Uncus wide at base, narrowed from base to basal 1/5, uniformly wide from basal 1/5 to basal 3/5, thereafter dilated elliptically, with a vertical semicircular sclerite near base; apex rounded, with a tuft of long setae. Gnathos weakly sclerotized laterally, just exceeding posterior margin of tegumen, invisible anteriorly. Tegumen widened medially; lateral arm narrow and short, shorter than median width, narrowed anteriorly. Valva triangular, narrow at base, widened to apex, with a small pad bearing several long setae at basal 1/3 on inner surface, with strong long setae distally; apex obliquely rounded, with a row of short setae arranged like a comb; costa narrow, lineate in distal half, with sparse long setae; ventral margin heavily sclerotized, forming a narrow band. Sacculus wide at base, slightly narrowed from base to apex; apex rounded, heavily sclerotized, covered with sparse setae. Saccus wide at base, narrowed to rounded apex; slightly shorter than uncus. Juxta slender, weakly arched. Phallus longer than valva, wide medially, with a large U-shaped sclerite distally.

***Female genitalia*** (Fig. 38). Papillae anales relatively short, rounded caudally, setose. Apophyses anteriores approximately 1/2 length of apophyses posteriores. Eighth sternal plate spiculate; posterior margin gently concave at middle, with long setae. Lamella antevaginalis trapezoidal. Ductus bursae membranous, narrower in posterior half, wide in anterior half, anteriorly with a round sclerotized area full of granules; ductus seminalis arising from middle of ductus bursae. Corpus bursae elliptical, slightly longer than ductus bursae, with dense denticles entirely, denticles larger from posterior 1/6 to middle, forming a conspicuous inverted U-shaped area.

##### Distribution.

Hainan (Bawangling, Jianfengling).

##### Etymology.

The specific epithet is derived from the Latin *pectinalis* (adj., comblike), referring to the valva with a row of setae along apex arranged like a comb.

#### Meleonoma hainanensis

Taxon classificationAnimaliaLepidopteraCosmopterigidae

Wang sp. nov.

23AC450B-AD66-5D59-A3B7-12208356D252

http://zoobank.org/91C8FDF5-D5E2-4698-B928-573D72EC106C

Figs 11 , 27

##### Type material.

China, Hainan: ***Holotype*** ♂, Mt. Diaoluo (18.73N, 109.87E), Lingshui, 980 m, 24.IV.2014, leg. TT Liu et al., slide No. YAH15508. ***Paratype***: 1♂, same data as holotype.

##### Diagnosis.

The new species can be distinguished from its congeners by the uncus with a sclerotized conic plate from middle of base reaching distal 1/3, and the valva distinctly angled at basal 1/3 on the ventral margin.

##### Description.

Adult (Fig. 11). Wingspan 14.5‒15.0 mm. Head with frons yellow on upper half, black on lower half; vertex blackish brown, yellow along dorsal margin of eyes and occiput. Labial palpus yellow; second segment mixed with dense black scales on outer surface, forming a black ring apically; third segment mixed with sparse black scales. Antenna with scape black, yellow along posterior margin; flagellum black annulated with yellow dorsally, yellow ventrally. Thorax blackish brown, edged with yellow scales; tegula blackish brown, tinged with yellow scales distally. Forewing blackish brown, with yellow and black scales; costal yellow spot situated before middle, crossing anterior margin of cell posteriorly, distal yellow spot at 1/4, sub-triangular, narrowed posteriorly, extending beyond anterior angle of cell, with a blackish brown dot at middle anteriorly; dorsal yellow spot diffused, ill-defined; cell with a black spot beyond basal 2/3 and at posterior angle respectively, both edged with yellow scales; plical spot black, at distal 2/5 of fold; terminal dots blackish brown interrupted by yellow scales, extending from distal part of costal margin through termen to before tornus; fringe blackish grey tinged with yellow. Hindwing and fringe deep grey. Legs yellow, with exception on ventral surface: foreleg with coxa greyish black, tarsus greyish black except yellow at apices of basal two tarsomeres, tibiae of fore- and midlegs greyish black, yellow apically, mid tarsus greyish black except yellow at apex of each tarsomere, hind tibia greyish black, tarsus mixed with sparse greyish black scales; all femora covered with dense greyish black scales.

***Male genitalia*** (Fig. 27). Uncus triangular, wide at base, narrowed to basal 2/3, then abruptly narrowed and sclerotized to hooked apex, edged with long setae; ventral surface with a sclerotized conic plate extending from middle of base to 2/3 length of uncus. Tegumen inverted U-shaped, widened medially; lateral arm narrowed anteriorly. Valva narrow at base, distinctly widened to basal 1/3, thereafter slightly narrowed to obtuse apex, setose; ventral margin heavily sclerotized along basal 1/3, forming a sclerotized band, distinctly angled at basal 1/3; costa straight, wide at base, narrowed distally, reaching beyond middle length of valva, lined with sparse setae; transtilla narrow, extending obliquely downward, not meeting medially. Sacculus wide at base, narrowed to basal 2/3; distal 1/3 sub-quadrate, heavily sclerotized, setose, obtuse at apex; dorsal margin concave at middle, ventral margin concave before distal 1/3. Saccus sub-triangular, wide at base, narrowed to narrowly rounded apex. Juxta V-shaped. Phallus slightly longer than valva, wide medially, with curved, irregularly wide belts distally.

**Female** unknown.

##### Distribution.

Hainan (Mt. Diaoluo).

##### Etymology.

The specific epithet is from the type locality, Hainan, China.

#### Meleonoma ornithorrhyncha

Taxon classificationAnimaliaLepidopteraCosmopterigidae

Wang sp. nov.

175CCC59-50FA-5084-92FA-749A3300F465

http://zoobank.org/89D979E4-ACBC-4B3A-9B97-93AF18FD019D

Figs 12 , 28 , 39

##### Type material.

China, Hainan: ***Holotype*** ♂, Tianchi (18.74N, 108.84E), Jianfengling, 1050 m, 30.IV.2013, leg. YH Sun et al., slide No. YAH15446. ***Paratypes*** (2♂1♀): 1♂1♀, 29‒30.IV.2013, other same data as holotype, slide No. ZXJ19027♀; 1♂, 770 m, Jianfengling, 30.V.2015, leg. PX Cong et al.

##### Diagnosis.

The new species can be distinguished from its congeners by the distally dilated valva with a beak-shaped process ventroapically, the U-shaped juxta with large spine-shaped lateral lobes sharp at apex, and the sacculus fused with the valva, and by the entirely sclerotized ductus bursae, and the two large signa of the corpus bursae each with a strong spine medially.

##### Description.

**Figures 12–19.**
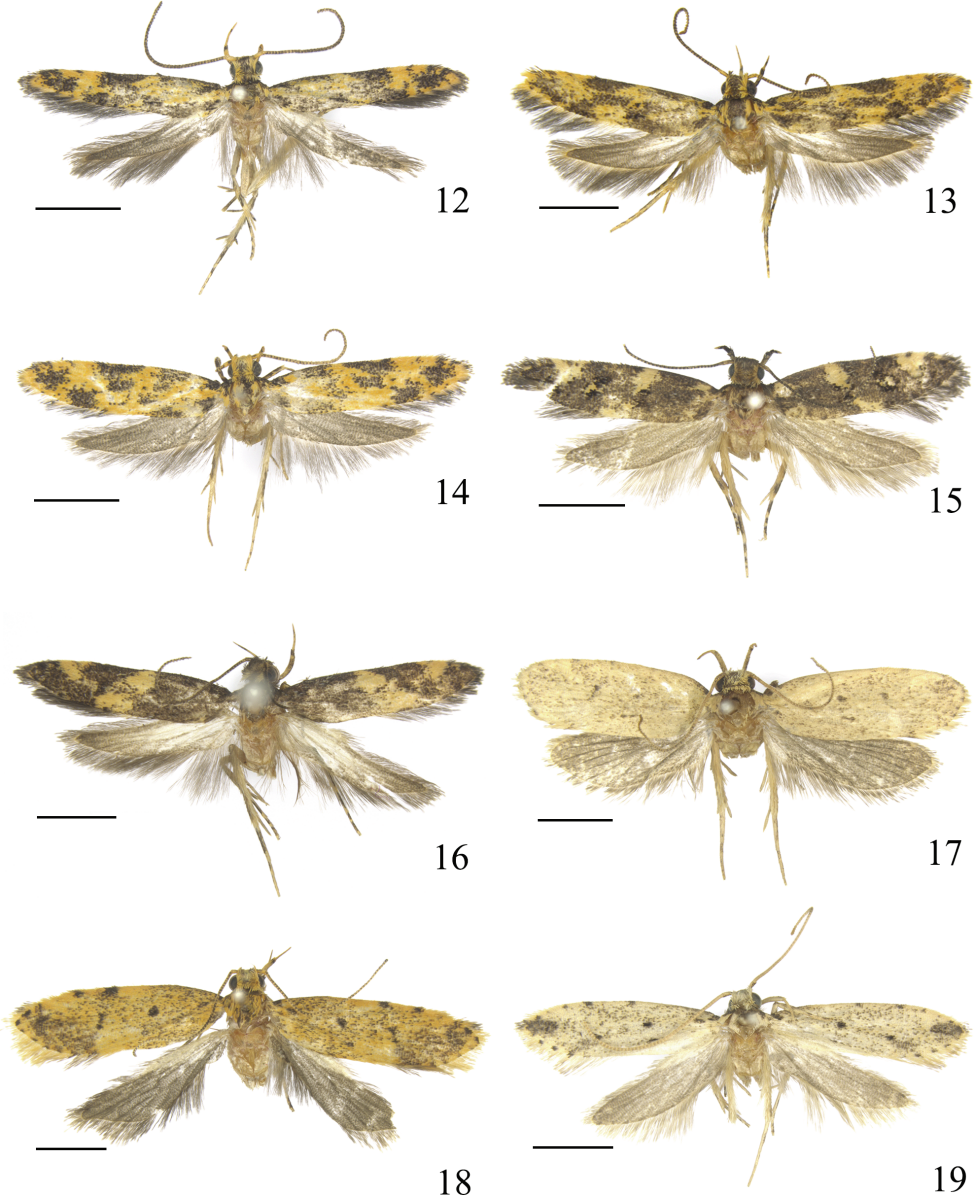
Male adults of *Meleonoma* spp. **12***M. ornithorrhyncha* sp. nov. **13***M. parilis* sp. nov. **14***M. quadritaeniata* sp. nov. **15***M. bidentata* sp. nov. **16***M. apicirectangula* sp. nov. **17***M. conica* sp. nov. **18***M. puncticulata* sp. nov. **19***M. robustispina* sp. nov. All holotypes. Scale bars: 2.0 mm.

Adult (Fig. 12). Wingspan 10.0‒11.0 mm. Head with frons yellow; vertex blackish brown, with yellow scales laterally. Labial palpus yellow; first segment black on outer surface; second segment with dense blackish brown scales in basal 3/4 on outer surface, with a black ring apically; third segment with a black dot medially on dorsal surface, nearly 2/3 as long as second segment. Antenna yellow; flagellum ringed with blackish brown. Thorax blackish brown, yellow at base laterally; tegula blackish brown on basal half, yellow on distal half. Forewing elongate narrow, apex pointed; ground color blackish brown, with an irregular longitudinal yellow stripe from base to middle; costal margin with median yellow spot placed before middle, diffused to preceding stripe posteriorly, distal yellow spot large, irregular, mixed with blackish brown scales, placed beyond distal 1/3, extending to posterior angle of cell posteriorly, with a blackish brown dot at middle anteriorly; small yellow spots running from apex along termen, last spot extending to distal spot of costal margin; dorsum with small yellow spot near base and at end of fold respectively; plical spot black, at distal 1/3 of fold; fringe blackish brown, with a pale yellow basal line. Hindwing and fringe greyish black. Legs yellow, with exception on ventral surface: fore coxa black, femur black; femora of mid- and hindlegs with scattered black scales, tarsi black except yellow at apices of basal three tarsomeres; hind tarsus black, yellow at apex of each tarsomere; all tibiae black except yellow apically.

**Figures 28–35.**
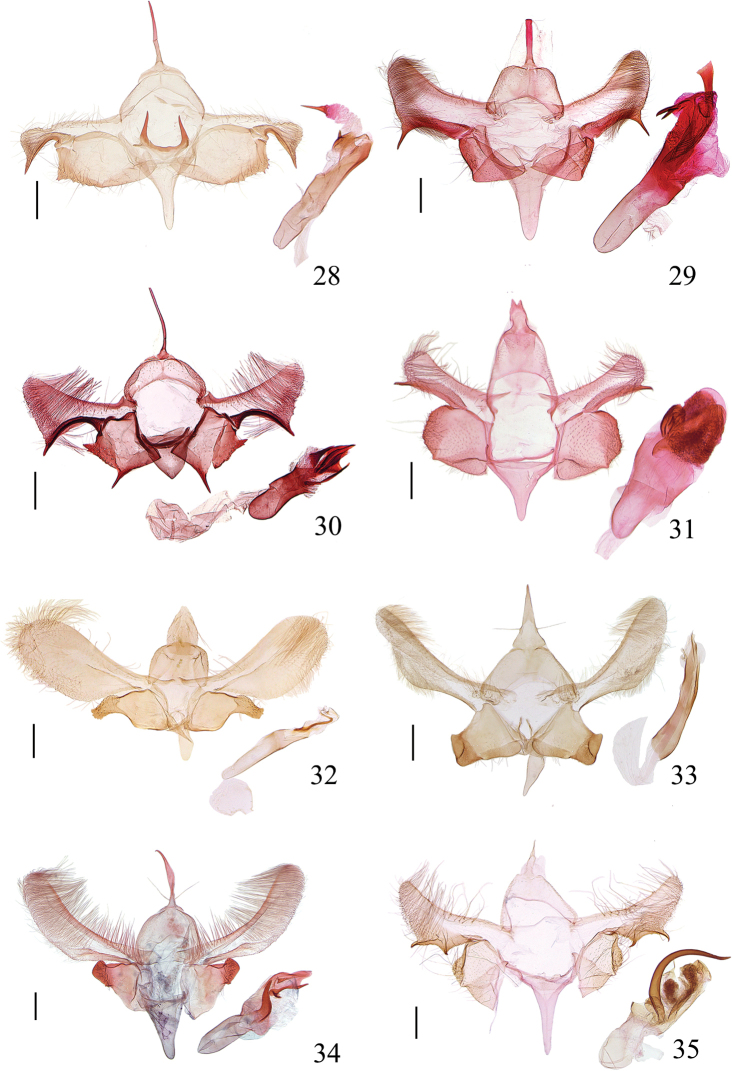
Male genitalia of *Meleonoma* spp. **28***M. ornithorrhyncha* sp. nov., slide No. YAH15446 **29***M. parilis* sp. nov., slide No. YAH15444 **30***M. quadritaeniata* sp. nov., slide No. YAH15450 **31***M. bidentata* sp. nov., slide No. ZXJ18110 **32***M. apicirectangula* sp. nov., LJ17529 **33***M. conica* sp. nov., slide No. LJ17553 **34***M. puncticulata* sp. nov., slide No. YAH15063 **35***M. robustispina* sp. nov., slide No. ZXJ19014. All holotypes. Scale bars: 0.2 mm.

***Male genitalia*** (Fig. 28). Uncus slender, clavate, pointed at apex. Gnathos relatively wide, sclerotized laterally, just exceeding posterior margin of tegumen, invisible anteriorly. Tegumen uniform except lateral arm slightly narrowed anteriorly. Valva uniform from base to beyond distal 1/3, birdhead-shaped distally, rounded dorsoapically, with a beak-shaped process ventroapically; costa banded, slightly concave at distal 1/4; transtilla slender, clavate, joined by membrane medially. Sacculus irregularly quadrangular, fused with valva, boundary narrowly banded; ventral margin heavily sclerotized, forming a sclerotized band, convex medially, with dense short setae in distal half, with several denticles distally; apex straight, heavily sclerotized, dentate ventroapically. Saccus wide at base, narrowed to middle; distal half uniformly narrow, apex rounded; same length as uncus. Juxta broad U-shaped; lateral arm slender, heavily sclerotized, spine-shaped, sharp at apex. Phallus longer than valva, membranous in part distally; with a sub-quadrate sclerite bearing a short apical spine; cornutus long, spine-shaped, placed in vesica.

***Female genitalia*** (Fig. 39). Papillae anales sub-quadrate, setose. Apophyses anteriores ca. 2/3 length of apophyses posteriores. Eighth sternal plate spiculate; posterior margin notched at middle, sparsely setose. Lamella antevaginalis concave medially on posterior margin, produced laterally. Antrum sub-trapezoidal, extended posterolaterally. Ductus bursae entirely sclerotized, shrunk laterally below antrum, slightly narrowed medially. Corpus bursae rounded, with rumples; with two large singa, each with a strong spine medially.

##### Distribution.

Hainan (Jianfengling).

##### Etymology.

The specific epithet is derived from the Latin *ornithorrhynchus* (adj., beak-like), referring to the distally birdhead-shaped valva with a beak-shaped process ventroapically.

#### Meleonoma parilis

Taxon classificationAnimaliaLepidopteraCosmopterigidae

Wang sp. nov.

E5FC024C-E0BF-595F-A72B-AD4CDA86CA98

http://zoobank.org/41EE2EA6-DC40-4C7F-9390-0C3DC9A10A8A

Figs 13 , 29 , 40

##### Type material.

China, Hainan: ***Holotype*** ♂, Forest Park (19.17N, 109.73E), Mt. Limu, 607 m, 24.VII.2014, leg. PX Cong et al., slide No. YAH15444. ***Paratypes*** (49♂8♀): 2♂1♀, 23‒24.VII.2014, other same data as holotype, slide No. YAH15503♀; 1♀, Bawangling, 650 m, 7.IV.2008, leg. BB Hu & HY Bai; 11♂4♀, Yinggezui, Yinggeling, 599 m, 27.VII‒1.VIII.2017, leg. X Bai et al.; 27♂1♀, Mt. Wuzhi, 738 m, 2‒3.III.2016, leg. QY Wang et al.; 1♂, Jianfengling, 940 m, 4.VI.2007, leg. ZW Zhang & WC Li; 1♂1♀, Tianchi, Jianfengling, 790 m, 1.IV.2008, leg. BB Hu & HY Bai; 1♂, Jianfengling, 1050 m, 27.IV.2014, leg. TT Liu et al.; 1♂, 15.VII.2015, 2♂, 5‒6.III.2016, Tianchi, Jianfengling, 787 m, QY Wang et al.; 3♂, Mingfenggu, Jianfengling, 954 m, 8‒9.VIII.2017, leg. X Bai et al.

##### Diagnosis.

The new species can be distinguished from its congeners in the male genitalia by the straightly uniform uncus with a truncate apex. It is similar to *M. quadritaeniata* sp. nov. in the male genitalia, but can be separated from the latter by the sacculus lacking a ventroapical process, the elongate saccus longer than the uncus, and the phallus with a wide banded plate distally; in *M. quadritaeniata*, the sacculus has a spine-shaped ventroapical process, the small saccus is shorter than the uncus, and the phallus has four narrowly banded sclerites distally. It is similar to *M. flavifasciana* Kitajima & Sakamaki, 2019 in the female genitalia, but can be distinguished by the lamella antevaginalis convex medially on the anterior margin, and the corpus bursae without a signum; whereas the lamella antevaginalis is concave medially on the anterior margin, and the corpus bursae has a signum in *M. flavifasciana* (Kitajima and Sakamaki 2019: 41, fig. 24).

##### Description.

Adult (Fig. 13). Wingspan 11.0‒12.0 mm. Head with frons blackish grey mixed with yellow scales; vertex blackish grey, yellow laterally. Labial palpus yellow; first and second segments mixed with dense blackish grey scales, with a black ring apically; third segment 2/3 length of second segment, blackish grey in basal 2/3. Antenna yellow; scape with scattered blackish grey scales; flagellum ringed with blackish grey. Thorax blackish grey, yellow laterally; tegula blackish grey basally, yellow distally. Forewing elongate-lanceolate, apex narrowly rounded; ground color orange-yellow; costal margin with four black spots: first one at base, rounded, extending to above fold posteriorly; second one horizontally narrow rectangular, from between basal 1/6 and 1/3 extending to above anterior margin of cell posteriorly; third one largest, parallelogram-shaped, from between 2/5 and distal 1/3 crossing anterior margin of cell posteriorly; fourth one small, at ca. distal 1/4, consisting of a few black scales; apex with a large black spot, diffused to costal margin and termen; tornal spot black, large, extending to posterior corner of cell; cell with a diffused black spot near base and a rectangular spot at ca. distal 1/3; plical spot black, large, at basal 2/3 of fold, diffused to discal spot anteriorly; dorsum greyish black along basal 2/3, forming a large stripe; fringe blackish grey, yellow basally. Hindwing and fringe deep grey, yellow basally. Legs orange-yellow, with exception on ventral surface: fore coxa blackish brown; tarsi of fore- and midlegs blackish brown, yellow at apices of basal two tarsomeres; hind tarsus blackish brown, yellow at apex of each tarsomere; all femora tinged with blackish brown scales, tibiae blackish brown, yellow apically.

***Male genitalia*** (Fig. 29). Uncus straight, uniform in width from near base to truncate apex. Gnathos sclerotized laterally, crossing anterior margin of tegumen, membranous anteriorly. Tegumen widened medially, concave anteriorly; lateral arm narrowed anteriorly, shorter than median width. Valva with basal half uniformly wide, widened from middle to apex, with a small setose pad at base; apex obtusely rounded, produced dorsoapically, with long setae; ventral margin heavily sclerotized, forming a wide band with a spine-shaped apical process ca. 2/5 length of uncus, directing downward, almost forming a right angle with ventral margin; costa widely banded, reaching pre-apex of valva; transtilla weakly sclerotized, indistinctly joined by membrane medially. Sacculus sub-rectangular, shorter than width, broadly overlapped ventrally; apex heavily sclerotized, with dense setae. Saccus wide at base, narrowed from base to rounded apex, slightly longer than uncus. Juxta slender, arched in C-shape. Phallus longer than valva, basal half uniformly wide, weakly sclerotized; distal half mostly membranous, with fine wrinkles; long and wide banded plate from before apex far exceeding apex, widened and truncate apically; cornuti being two large spines joined at base.

***Female genitalia*** (Fig. 40). Papillae anales sub-quadrate, setose. Apophyses posteriores approximately 3.0 × length of apophyses anteriores. Eighth sternal plate spiculate, emarginate at middle on posterior margin, with sparse setae, straight on anterior margin. Lamella antevaginalis U shaped; lateral arm widened laterally, forming two large fan-shaped plates, arched and serrate on inner margin, obliquely straight on outer margin, convex on anterior margin. Antrum quadrate. Ductus bursae membranous; ductus seminalis arising from ductus bursae, dilated basally. Corpus bursae almost as long as ductus bursae, rounded, densely spiculate; signum absent.

**Figures 36–41.**
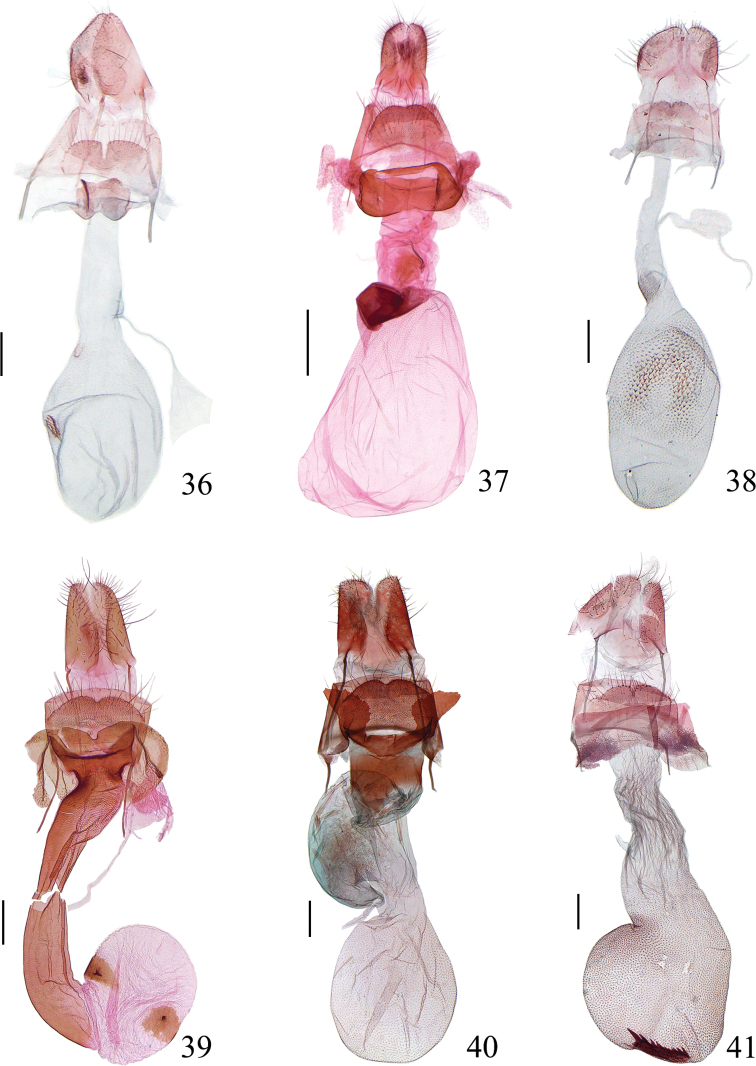
Female genitalia of *Meleonoma* spp. **36***M. rostellata* sp. nov., slide No. ZXJ18410 **37***M. magnidentata* sp. nov., slide No. ZXJ18261 **38***M. pectinalis* sp. nov., slide No. YAH15095 **39***M. ornithorrhyncha* sp. nov., slide No. ZXJ19027 **40***M. parilis* sp. nov., slide No. YAH15503 **41***M. bidentata* sp. nov., slide No. YAH15442. All paratypes. Scale bars: 0.2 mm (**36, 38–41**); 0.5 mm (**37**).

##### Distribution.

Hainan (Bawangling, Jianfengling, Mt. Limu, Mt. Wuzhi, Yinggeling).

##### Etymology.

The specific epithet is derived from the Latin *parilis* (adj., evenly wide), referring to the evenly wide uncus.

#### Meleonoma quadritaeniata

Taxon classificationAnimaliaLepidopteraCosmopterigidae

Wang sp. nov.

04C50C57-C70F-56B1-9E72-4BBDEBC98C81

http://zoobank.org/846A642E-69B2-4D88-BF6F-927BBEAD770C

Figs 14 , 30

##### Type material.

China, Hainan: ***Holotype*** ♂, Mt. Wuzhi (18.91N, 109.68E), 710 m, 21.IV.2014, leg. TT Liu et al., slide No. YAH15450. ***Paratypes***: 2♂, Mt. Wuzhi, 740 m, 12‒14.IV.2009, leg. Q Jin & BB Hu.

##### Diagnosis.

The new species can be distinguished from its congeners by the phallus with four narrowly band-shaped sclerites in distal 1/3. It is similar to *M. parilis* sp. nov., and the differences between them can be found in the diagnosis of the preceding species.

##### Description.

Adult (Fig. 14). Wingspan 10.0‒11.0 mm. Head orange, frons with blackish brown scales, vertex with scales tipped blackish brown. Labial palpus orange; first and second segments with black scales on outer surface; second segment with a black ring apically; third segment tinged with brown scales, nearly 2/3 length of second segment. Antenna yellow; scape black on basal half dorsally; flagellum ringed with blackish brown on dorsal surface. Thorax black medially, yellow laterally; tegula black basally, yellow distally. Forewing orange-yellow; costal margin with four black spots: first spot at base, sub-rectangular, extending to fold posteriorly; second spot from beyond basal 1/4 diffused and narrowed to middle of cell posteriorly; third spot from basal 1/3 widened to before distal 1/3; fourth spot small, placed at distal 1/5, consisting of several black scales; apex with a large diffused black spot, anteriorly diffused to costal margin; cell with a diffused black spot at middle and a rectangular black spot at distal 1/3, the latter below third costal spot; plical spot at middle of fold, diffused to second costal spot; tornal spot large, equally sized with apical spot; dorsum with black spot at base and before middle respectively; fringe greyish blackish except yellow basally. Hindwing and fringe blackish grey. Legs yellow, with exception on ventral surface: fore- and midlegs scattered with dense black scales, tibiae of fore- and midlegs yellow apically, tarsi of fore- and midlegs yellow at apices of basal two tarsomeres, hindleg whitish yellow mixed with black scales.

***Male genitalia*** (Fig. 30). Uncus extremely long and slender, slightly wide at base, pointed at apex. Gnathos sclerotized laterally, just exceeding posterior margin of tegumen, invisible anteriorly. Tegumen uniformly wide medially; lateral arm narrowed anteriorly. Valva wide at base, narrowed to middle, widened from middle to apex; apex obliquely obtuse, produced dorsoapically, with dense fine setae; ventral margin heavily sclerotized, forming a wide band, concave near base, produced to a strong spine directing exceeding apex of valva apically; costa wide basally, narrowed to middle, projected triangularly near base; transtilla short and stout. Sacculus sub-quadrate, dorsal margin heavily sclerotized, sinuate; ventral margin heavily sclerotized, forming a wide band with a strong spine-shaped apical process; apex heavily sclerotized, truncate, with a large tooth dorsoapically. Vinculum extended anteriorly, forming a small saccus. Juxta slightly arched. Phallus nearly as long as valva; basal 3/5 sclerotized, tubular; distal 2/5 membranous, with four narrowly banded sclerites pointed apically.

**Female** unknown.

##### Distribution.

Hainan (Mt. Wuzhi).

##### Etymology.

The specific epithet is derived from the Latin *quadri*- (of four) and *taeniatus* (adj., banded), referring to the four banded plates of the phallus.

### The *annulignatha* species group

#### Meleonoma bidentata

Taxon classificationAnimaliaLepidopteraCosmopterigidae

Wang sp. nov.

21DFC906-8D83-5CAD-8C57-2A11EC8FE49D

http://zoobank.org/16D5C9E5-5B92-4CD1-935B-64F831849121

Figs 15 , 31 , 41

##### Type material.

China, Hainan: ***Holotype*** ♂, Bawangling (19.10N, 109.11E), 245 m, 8.V.2013, leg. YH Sun et al., slide No. ZXJ18110. ***Paratypes*** (8♂2♀): 3♂1♀, 7‒9.V.2013, other same data as holotype, slide No. YAH15442♀; 1♂, Bawangling, 28.VII.2013, leg. HL Yu & KL Liu; 2♂, 146 m, 1♀, 225 m, 13–14.VIII.2017, Bawangling, leg. X Bai et al.; 2♂, Lemei, Dongfang, 81 m, 3.I.2018, leg. MJ Qi & S Yu.

##### Diagnosis.

The new species can be distinguished by the uncus with two denticles apically and the medially widened tegumen with slender lateral arm slightly extending inward anteriorly; and by the entirely spiculate corpus bursae with an elongate dentate signum at bottom.

##### Description.

Adult (Fig. 15). Wingspan 10.0‒11.0 mm. Head yellow; vertex blackish brown, lateral scales yellow basally. Labial palpus yellow; first and second segments with dense blackish grey scales on outer surface; second segment inflated at apex by rough scales; third segment shorter than 1/2 length of second segment, greyish black on basal half. Antenna with scape yellowish brown on dorsal surface, yellow on ventral surface; flagellum yellow alternated with greyish black. Thorax and tegula greyish black. Forewing dark brown, with diffused yellow spot near base below costal margin and at base of dorsum respectively; costal margin with median yellow spot rounded, from before middle reaching middle of cell posteriorly, edged with sparse black scales, distal yellow spot larger, inverted triangular, from distal 1/4 extending ventrad crossing middle of wing, with a small black dot at middle anteriorly; cell with a black spot beyond middle, anteriorly touching median spot of costal margin, with a large diffused black spot at outer margin, interrupted by a yellow spot; plical spot black, placed at distal 2/5 of fold, bordered by a yellow spot at outside; yellow dorsal spot rounded, at end of fold; fringe greyish black, tinged with yellow basally. Hindwing and fringe grey. Legs yellow, with exception on ventral surface: fore coxa with scattered blackish brown scales, tarsi of fore- and midlegs blackish brown, yellow at apices of basal two and apical one tarsomeres, hind tarsus with basal three tarsomeres blackish brown except yellow at apex of each tarsomere; all femora with dense blackish brown scales, tibiae blackish brown except yellow apically.

***Male genitalia*** (Fig. 31). Uncus narrowed sub-basally, widened at basal 2/5, thereafter slightly narrowed to apex; apex bifurcated, forming two denticles apically. Gnathos sclerotized laterally, membranous anteriorly. Tegumen widened medially; lateral arm slender, slightly extending inward anteriorly. Valva wide at base, weakly narrowed medially, gradually widened to apex; apex broadly rounded, with large dense setae; ventral margin heavily sclerotized, forming a sclerotized band with an apical spine exceeding apex of valva; costa narrowly banded, reaching apex of valva, with long setae; transtilla bilobed: dorsal lobe narrowly extended to pointed apex, indistinctly connected by membrane, ventral lobe shorter than dorsal lobe, rounded at apex. Sacculus sub-quadrate; apex heavily sclerotized, forming a setose narrow band; dorsal margin narrowly sclerotized. Saccus wide at base, narrowed to before obtuse apex; longer than uncus. Juxta broadly arched. Phallus stout, almost twice length of valva, narrow at base, widened medially; distal half membranous, full of fine rumples, with a wide sclerotized band from middle curved spirally to near apex.

***Female genitalia*** (Fig. 41). Papillae anales rectangular, setose. Apophyses posteriores approximately 3.0 × as long as apophyses anteriores. Eighth sternal plate spiculate; posterior margin with long setae, notched at middle. Lamella antevaginalis straight posteriorly and laterally, slightly concave medially on anterior margin, with two reticulate areas anterolaterally. Antrum band-shaped, arched backwards medially. Ductus bursae membranous, posterior 3/4 narrower, wrinkled; anterior 1/4 wide, with dense granules; ductus seminalis arising from anterior 1/3 of ductus bursae. Corpus bursae half as long as ductus bursae, entirely spiculate, irregular in shape; signum elongate rectangular, with numerous teeth and one large apical spine, placed at bottom of corpus bursae.

##### Distribution.

Hainan (Bawangling, Dongfang).

##### Etymology.

The specific epithet is derived from the Latin *bidentatus* (adj., bidentate), referring to the two apical denticles of the uncus.

### The *fasciptera* species group

#### Meleonoma apicirectangula

Taxon classificationAnimaliaLepidopteraCosmopterigidae

Wang sp. nov.

E7F0F831-8886-52B8-B7BF-9FACEB178AE6

http://zoobank.org/293594B3-C2B2-4922-8899-29F7EF4811E3

Figs 16 , 32

##### Type material.

China, Hainan: ***Holotype*** ♂, Bawangling (19.07N, 109.03E), 650 m, 7.IV.2008, leg. BB Hu & HY Bai, slide No. LJ17529. ***Paratypes*** (3♂): 1♂, Bawangling, 1000 m, 9.IV.2008, leg. BB Hu & HY Bai; 1♂, Hongxin, Yuanmen, Baisha, 430 m, 16.IV.2014, leg. TT Liu et al.; 1♂, Mt. Wuzhi, 738 m, 2.XI.2016, leg. X Bai et al.

##### Diagnosis.

The new species is similar to *M. neargometra* (Wang, 2003) in the male genitalia. It can be separated from the latter by the transtilla pointed at apex and the sacculus produced to a sub-rectangular process dorsoapically. In *M. neargometra*, the transtilla is rounded at apex and the sacculus is produced to a sub-triangular process dorsoapically (Wang 2003: 202, fig. 9).

##### Description.

Adult (Fig. 16). Wingspan 13.0–14.0 mm. Head with frons yellowish white mixed with blackish brown, vertex blackish brown. Labial palpus yellow; distal half of second segment mixed with dense black scales on outer surface, forming a black ring at apex; third segment mixed with dense black scales from distal half to before apex ventrally. Antenna blackish brown; scape mixed with yellow; flagellum annulated with yellow ventrally. Thorax and tegula blackish brown. Forewing blackish brown, tinged with pale yellow to yellow scales; median fascia yellow, with blackish brown scales medially, extending from basal 2/5 of costal margin obliquely outward to around tornus, widened posteriorly; costal spot yellow, inverted triangular, edged with sparse blackish brown scales, extending to outer margin of cell posteriorly; fringe blackish brown. Hindwing and fringe greyish brown. Legs yellow, with exception on ventral surface: fore coxa blackish brown; femora of fore- and midlegs with dense blackish brown scales, hind femur with sparse blackish brown scales; tarsi of fore- and midlegs blackish brown, yellow at apices of basal two and apical one tarsomeres, hind tarsus with basal three tarsomeres blackish brown except yellow apically; all tibiae blackish brown except yellow apically.

***Male genitalia*** (Fig. 32). Uncus broad at base, narrowed to rounded apex, with long setae mediolaterally. Tegumen narrowed medially; lateral arm narrowed anteriorly. Valva narrow at base, widened from base to basal 1/3, uniformly wide from basal 1/3 to pre-apex, rounded at apex; costa band-shaped, uniformly wide, reaching distal 1/3 of valva, with sparse long setae; transtilla heavily sclerotized, uniformly wide except pointed apically, joined by membrane medially; sclerotized fold from base of ventral margin extending to middle of valva, parallel with costa. Sacculus sub-quadrate, dorsoapically produced to a heavily sclerotized sub-rectangular process, densely setose, straight at apex, sinuate dorsally; sclerotized on dorsal and ventral margins. Saccus wide at base, slightly narrowed to rounded apex. Juxta arched. Phallus shorter than valva, widened medially; distal half membranous, with a curved slender belt as long as 2/5 length of phallus.

**Female** unknown.

##### Distribution.

Hainan (Baisha, Bawangling, Mt. Wuzhi).

##### Etymology.

The specific epithet is derived from the Latin *apic*- (adj., apical) and *rectangulus* (adj., rectangular), referring to the shape of the apical process of the sacculus.

### The *puncticulata* species group

#### Meleonoma conica

Taxon classificationAnimaliaLepidopteraCosmopterigidae

Wang sp. nov.

CAC382B2-D1F5-53F4-8AE9-CE707FEF73B1

http://zoobank.org/6FFC20C6-9F39-4006-8992-A70C559EBE9C

Figs 17 , 33

##### Type material.

China, Hainan: ***Holotype*** ♂, Datian (19.11N, 108.79E), Dongfang, 56 m, 7.VI.2018, leg. P Liu et al., slide No. LJ17553.

##### Diagnosis.

The new species is similar to *M. leishana* (Wang, 2006), *M. stica* (Wang, 2006) and *M. puncticulata* sp. nov. in the forewing patterns. It can be distinguished from *M. leishana* and *M. stica* by the uncus longer than the saccus and the phallus without sclerotized belts distally; in *M. leishana* (Wang, 2006) (Wang 2006a: 131, fig. 216) and *M. stica* (Wang, 2006) (Wang 2006b: 25, fig. 15), the uncus is shorter than the saccus and the phallus has sclerotized belts distally. It can be separated from *M. puncticulata* by the uncus tapered from base to apex, the relatively narrower and shorter saccus ca. 3/5 the length of the uncus, and the phallus without cornutus; in *M. puncticulata*, the uncus is widened from base to middle, thereafter narrowed to apex, the saccus is almost as long as the uncus, and the phallus has a strong cornutus.

##### Description.

Adult (Fig. 17). Wingspan 14.5 mm. Head yellow. Labial palpus yellow; second segment with blackish brown scales dorsally, forming a dark dot before apex; third segment with sparse blackish brown scales dorsally. Antenna yellow, flagellum annulated with brown (worn). Thorax yellow (worn); tegula yellow, blackish brown at base. Forewing yellow, with blackish brown scales; costal margin with blackish brown dot at base, beyond middle and at distal 1/3 respectively; cell with a blackish brown spot beyond middle and at outer margin respectively; plical spots blackish brown, small, rounded; terminal dots running from distal part of costal margin along termen to tornus, evenly spaced; fringe yellow. Hindwing and fringe grey. Legs yellow, with exception on ventral surface: fore- and midlegs blackish brown, tibia of midleg yellow apically, tarsus of midleg yellow at apex of each tarsomere, hindleg covered with blackish brown scales.

***Male genitalia*** (Fig. 33). Uncus conic, wide at base, distinctly tapered from base to apex, with a single long seta at basal 1/3 laterally. Tegumen slightly widened medially; lateral arm uniformly wide, obtuse anteriorly. Valva with basal 1/3 narrow, wide and subparallel medially, slightly narrowed from distal 1/5 to rounded apex, setose, with a densely setose pad at base; ventral margin weakly sclerotized, concave basally; costa with basal half uniformly wide, distal half narrowed to before apex; transtilla short, not meeting medially. Sacculus wide at base, narrowed from base to rounded apex; distal 1/3 heavily sclerotized, setose, overlapped with an ovate plate; ventral margin heavily sclerotized from base to distal 1/3, forming a wide band, with long setae. Saccus ca. 3/5 length of uncus, wide at base, narrowed from base to rounded apex. Juxta U-shaped; lateral lobe slender. Phallus approximately 4/5 length of valva, with lineate ridges distally; cornutus absent.

**Female** unknown.

##### Distribution.

Hainan (Dongfang).

##### Etymology.

The specific epithet is derived from the Latin *conicus* (adj., conic), referring to the shape of the uncus in the male genitalia.

#### Meleonoma puncticulata

Taxon classificationAnimaliaLepidopteraCosmopterigidae

Wang sp. nov.

260AA0F6-A0F2-51C6-8102-779C2FB56A61

http://zoobank.org/65AACDFC-71DA-41B5-ADD6-03CFCB116E84

Figs 18 , 34

##### Type material.

China, Hainan: ***Holotype*** ♂, Mt. Diaoluo (18.73N, 109.87E), 980 m, 23. IV.2014, leg. TT Liu et al., slide No. YAH15063. ***Paratypes*** (3♂): 1♂, Mt. Diaoluo, 940 m, 2.VI.2007, leg. ZW Zhang & WC Li; 1♂, Mt. Limu, 640 m, 1.V.2014, leg. TT Liu et al.; 1♂, Jianfengling, 940 m, 4.VI.2007, leg. ZW Zhang & WC Li.

##### Diagnosis.

The new species is similar to *M. leishana* (Wang, 2006) and *M. stica* (Wang, 2006) superficially. It differs from the latter two species by the saccus almost as long as the uncus and the valva evenly wide distally; whereas the saccus is distinctly longer than the uncus and the valva is widened distally before apex in *M. leishana* (Wang 2006a: 131, fig. 216) and *M. stica* (Wang 2006b: 25, fig. 15). The new species is similar to *M. conica* sp. nov., and the differences between them are stated in the preceding species.

##### Description.

Adult (Fig. 18). Wingspan 12.0‒13.0 mm. Head yellow, vertex with brown scales, frons pale yellow. Labial palpus yellow; second segment with sparse blackish brown scales on outer surface, with a blackish brown ring before apex; third segment slightly shorter than second segment, blackish brown ventrally. Antenna with scape yellow, with blackish brown scales on anterior margin; flagellum blackish brown annulated with yellow dorsally, yellow ventrally. Thorax and tegula yellow mixed with sparse blackish brown scales. Forewing pale yellow to orange-yellow, with blackish brown scales; costal margin with blackish brown dot at base, at basal 3/5 and distal 1/4 respectively; discal and plical spots blackish brown, small and rounded; cell with a round blackish brown at middle, with a transversely elongate spot before posterior angle of cell; dense blackish brown scales from apex along termen to tornus, forming an apical patch; terminal dots blackish brown, running from apex through termen to tornus; fringe yellow except blackish grey on distal part of costal margin and around tornus. Hindwing and fringe greyish brown. Legs yellow, with exception on ventral surface: foreleg blackish brown except coxa yellow mixed with blackish brown, tarsus yellow at apices of basal two tarsomeres; midleg with blackish brown scales on femur, tibia blackish brown except yellow apically, tarsus blackish brown except yellow at apices of basal two tarsomeres; hindleg tinged with blackish brown scales on tibia and tarsus.

***Male genitalia*** (Fig. 34). Uncus elongate, narrow at base, widened from base to middle, thereafter narrowed to pointed apex, with a single long seta laterally near base. Gnathos very short, only distinct basally. Tegumen widened medially; lateral arm short, slightly narrowed anteriorly. Valva elongate, widened from base to basal 1/3, subparallel from basal 1/3 to rounded apex, setose; ventral margin with basal 1/3 sclerotized, forming a narrow band; costa wide at base, narrowed to before apex, with stout setae in basal half; transtilla expanded, with dense long setae. Sacculus wide at base, base 3.0 × width of apex, narrowed from base to before distal 1/3; distal part heavily sclerotized, setose, quadrate, truncate apically; dorsal margin sinuate, sclerotized, concave before apex; ventral margin normal, not sclerotized. Saccus almost as long as uncus, wide at base, narrowed to rounded apex. Juxta slender, arched. Phallus approximately 2/3 length of valva; basal 2/5 tubular, sclerotized, distal 3/5 membranous except sclerotized in part, with a small sclerite and indistinct folds distally; curved, sclerotized belt from ca. distal 1/4 exceeding apex of phallus, dilated and with a small sclerite basally; cornutus large and stout, spine-shaped, curved at base, extending from beyond basal 2/5 to before distal 1/4.

**Female** unknown.

##### Distribution.

Hainan (Jianfengling, Mt. Diaoluo, Mt. Limu).

##### Etymology.

The specific epithet is derived from the Latin *puncticulatus* (adj., having dots), referring to the forewing with several blackish brown dots.

#### Meleonoma robustispina

Taxon classificationAnimaliaLepidopteraCosmopterigidae

Wang sp. nov.

C27038AD-C0FC-572A-A0CF-056AAF828DB9

http://zoobank.org/B09235D7-D563-413A-8998-852710040EC8

Figs 19 , 35

##### Type material.

China, Hainan: ***Holotype***: ♂, Mt. Limu (19.17N, 109.73E), 632 m, 29.VI.2015, leg. QY Wang et al., slide No. ZXJ19014. ***Paratype***: 1♂, Yinggezui, Yinggeling, 599 m, 1.VIII.2017, leg. X Bai et al.

##### Diagnosis.

The new species can be distinguished from its congeners by the ventral band of the valva widened distally and having two spines apart from each other, the sacculus produced to a short semielliptical process apically, and the phallus with a large spine-shaped cornutus more than 1/2 length of the phallus.

##### Description.

Adult (Fig. 19). Wingspan 11.0‒13.0 mm. Head yellowish white. Labial palpus pale yellow; second segment with blackish brown scales on outer surface, with a black ring apically; third segment with blackish brown scales on dorsal surface. Antenna yellow; scape with blackish brown scales on posterior margin; flagellum annulated with greyish brown on dorsal surface. Thorax pale yellow except black at base; tegula black basally, pale yellow distally. Forewing pale yellow, with scattered blackish brown scales, with blackish brown spot at base and between Sc and fold respectively; costal margin with four blackish brown dots spaced from before middle to pre-apex, becoming smaller; apex with an elliptical blackish brown spot; termen with three blackish brown dots evenly spaced from below apical spot; cell with a small rounded blackish brown spot before posterior angle of cell; plical spot blackish brown, placed at middle of fold; fringe pale yellow, tinged with blackish brown on extension of apical spot, or blackish brown tinged with yellow except yellow on distal part of costal margin. Hindwing and fringe grey. Legs yellow, with exception on ventral surface: foreleg black, coxa yellow, tibia yellow apically, tarsus yellow at apices of basal two tarsomeres; mid tibia black, yellow apically, tarsus black except yellow at apices of basal two and apical one tarsomeres; hindleg mixed with sparse black scales on tibia and tarsus.

***Male genitalia*** (Fig. 35). Uncus with basal half evenly wide, distal half narrowed to pointed apex. Tegumen U-shaped, widened medially, sclerotized along outer and inner margins; lateral arm uniformly narrow. Gnathos very weak laterally, just exceeding posterior margin of tegumen, invisible anteriorly. Valva narrow at base, widened medially, thereafter narrowed to rounded apex, with long setae; ventral margin heavily sclerotized, forming a band widened distally, with two spines apart from each other: short preapical spine directing downward and curved inward, longer apical spine extending outward and exceeding ventroapical corner; costa slightly concave medially, with sparse long setae in distal 2/3; transtilla slender, meeting medially. Sacculus irregularly shaped, with a sclerotized narrow edge dorsally, produced to a setose, semielliptical process apically; ventral margin overlapped triangularly. Saccus longer than twice length of uncus, wide at base, narrowed to rounded apex. Juxta widely arched. Phallus slightly longer than valva; basal 2/5 tubular, weakly sclerotized; distal 3/5 partly membranous, wrinkled, with a sclerotized band from middle to pre-apex, ending with a spine; cornutus large spine-shaped, more than 1/2 length of the phallus, strongly arched medially, narrowed to pointed apex, extending from middle to beyond apex of phallus apically.

**Female** unknown.

##### Distribution.

Hainan (Mt. Limu, Yinggeling).

##### Etymology.

The specific epithet is derived from the Latin *robustispinus* (adj., having strong spines), referring to the strong spine-shaped cornutus of the phallus in the male genitalia.

### List of described species of *Meleonoma* in Hainan Island

#### The *segregnatha* species group

***Meleonoma apicispinata* Wang, 2016**

*Meleonoma apicispinata* Wang, 2016, In: Yin and Wang 2016a: 26.

**Distribution.** Hainan (Dongfang, Jianfengling, Mt. Limu, Mt. Wuzhi, Yinggeling).

***Meleonoma liui* (Wang, 2006)**

*Cryptolechia liui* Wang, 2006a: 132.

*Meleonoma liui*: Wang et al., 2020: 383.

**Distribution.** Hainan (Baisha, Danzhou, Jianfengling, Mt. Limu, Mt. Wuzhi, Yinggeling, Qixianling).

#### The *facialis* species group

***Meleonoma facialis* Li & Wang, 2002**

*Meleonoma facialis* Li & Wang, 2002: 230.

**Distribution.** Hainan (Bawangling, Dongfang, Duowenling, Gaoshanling, Yinggeling).

***Meleonoma polychaeta* Li, 2004**

*Meleonoma polychaeta* Li, 2004, In: Li & Wang, 2004: 35.

**Distribution.** Hainan (Mt. Wuzhi).

#### The *malacobyrsa* species group

***Meleonoma microbyrsa* (Wang, 2003)**

*Cryptolechia microbyrsa* Wang, 2003: 198.

*Meleonoma microbyrsa*: Wang et al., 2020: 382.

**Distribution.** Hainan (Bawangling, Baisha, Dongfang, Duowenling, Gaoshanling, Jianfengling, Mt. Diaoluo, Mt. Limu, Mt. Wolong, Mt. Wuzhi, Qixianling, Yinggeling).

## Supplementary Material

XML Treatment for Meleonoma apicicurvata

XML Treatment for Meleonoma linearis

XML Treatment for Meleonoma rostellata

XML Treatment for Meleonoma magnidentata

XML Treatment for Meleonoma bicuspidata

XML Treatment for Meleonoma latiunca

XML Treatment for Meleonoma pectinalis

XML Treatment for Meleonoma hainanensis

XML Treatment for Meleonoma ornithorrhyncha

XML Treatment for Meleonoma parilis

XML Treatment for Meleonoma quadritaeniata

XML Treatment for Meleonoma bidentata

XML Treatment for Meleonoma apicirectangula

XML Treatment for Meleonoma conica

XML Treatment for Meleonoma puncticulata

XML Treatment for Meleonoma robustispina
